# Bugs in Transition: The Dynamic World of *Wolbachia* in Insects

**DOI:** 10.1371/journal.pgen.1004069

**Published:** 2013-12-26

**Authors:** Wolfgang J. Miller

**Affiliations:** Department of Cell and Developmental Biology, Medical University of Vienna, Vienna, Austria; Fred Hutchinson Cancer Research Center, United States of America

The availability of Next Generation Sequencing tools has uncovered an unexpected and highly complex universe of hidden microbial passengers that are transiently or permanently associated with their hosts, hereafter called symbionts. Under natural conditions, these symbionts are often tightly controlled to occur at low densities and/or are restricted to special tissues by intrinsic and extrinsic factors, such as their own replication capacity, nutrition, and stress, as well as host immune competence.

One of the main questions of current symbiosis research is, however, to what extent these microbial passengers affect host phenotypes such as fitness, fecundity, pathogen resistance, or even behavior. In this issue of *PLOS Genetics*, Luis Teixeira and colleagues [1] have, very elegantly, started to answer some of these essential questions in symbiosis research. The focus of their research is on intracellular bacteria belonging to the genus *Wolbachia*, one of the most intensively studied symbionts. *Wolbachia* are found in up to 70% of insect species and in many terrestrial arthropods, and are vertically transmitted with the egg from an infected female to her progeny. In order to enhance their spreading success throughout host populations, *Wolbachia* can manipulate host reproductive biology by acting as a reproductive parasite, enhancing fitness and fecundity of infected females, enabling them to outcompete uninfected females rapidly in nature [2].

But what happens to the infection as soon as the majority of individuals within a host population are already infected and the spreading of the bacteria comes to a stop? In some cases it has been shown that, in their evolutionary past, *Wolbachia* have changed their phenotype from reproductive parasitism to obligate mutualism, where the symbiont takes on essential host functions for, e.g., oogenesis, nutrition, or even mate recognition [3]–[5]. Which kind of extrinsic and/or intrinsic factors trigger phenotypic transitions, and what is their genetic basis? In a recent study it was shown that within only 20 years in nature, *Wolbachia* can transform from a costly reproductive parasite into a mutualist by enhancing fecundity of infected *Drosophila simulans* females [6].

One of the most puzzling observations, however, is the worldwide replacement during the 20th century of one ancestral *Wolbachia* strain named wMelCS with the closely related variant wMel in *Drosophila melanogaster*, the top genetic model system for studying insect host-symbiont interactions [7], [8]. Although neither *Wolbachia* strain causes any significant level of reproductive parasitism in its respective native host, either in the laboratory or in nature, both persist globally in *D. melanogaster* at high frequencies worldwide, suggesting they serve some host functions. Moreover, two independent studies have shown that both *Wolbachia* strains confer significant virus protection [9], [10]. Impressively, *D. melanogaster Wolbachia* also protects artificially transinfected mosquitos that do not naturally carry *Wolbachia* from pathogenic RNA viruses, such as Dengue and Chikungunya [11]. A third *Wolbachia* strain of *D. melanogaster* that provides even stronger virus resistance is wMelPop, a pathogenic *Wolbachia* variant that originally emerged after radiation mutagenesis in the laboratory. This virulent *Wolbachia* strain has recently become a major research focus because it overreplicates massively in somatic host tissues (it is named “popcorn” due to the popcorn-like appearance of infected cells in adult tissues) and hence significantly shortens the lifespan of its host [12]. After transferring wMelPop into the main vector of dengue fever *Aedes aegypti*, it was shown that the life-shortening effect of wMelPop is also manifest in this medically important insect system [13]. The combination of virus protection, life shortening, and the capacity of maternal spreading render *D. melanogaster Wolbachia* strains prime candidates for biologically sound insect pest control strategies. Besides their impressive applied capacities for arthropod-borne disease control, however, we currently lack understanding on the following main questions: what are the genotypic and phenotypic differences between the three strains, and why did the wMel strain replace wMelCS globally in the recent past? A better understanding of their genetic basis and their short-term cost–benefit dynamics will be pivotal for further *Wolbachia*-applied studies in heterologous pest systems.

**Figure 1:**
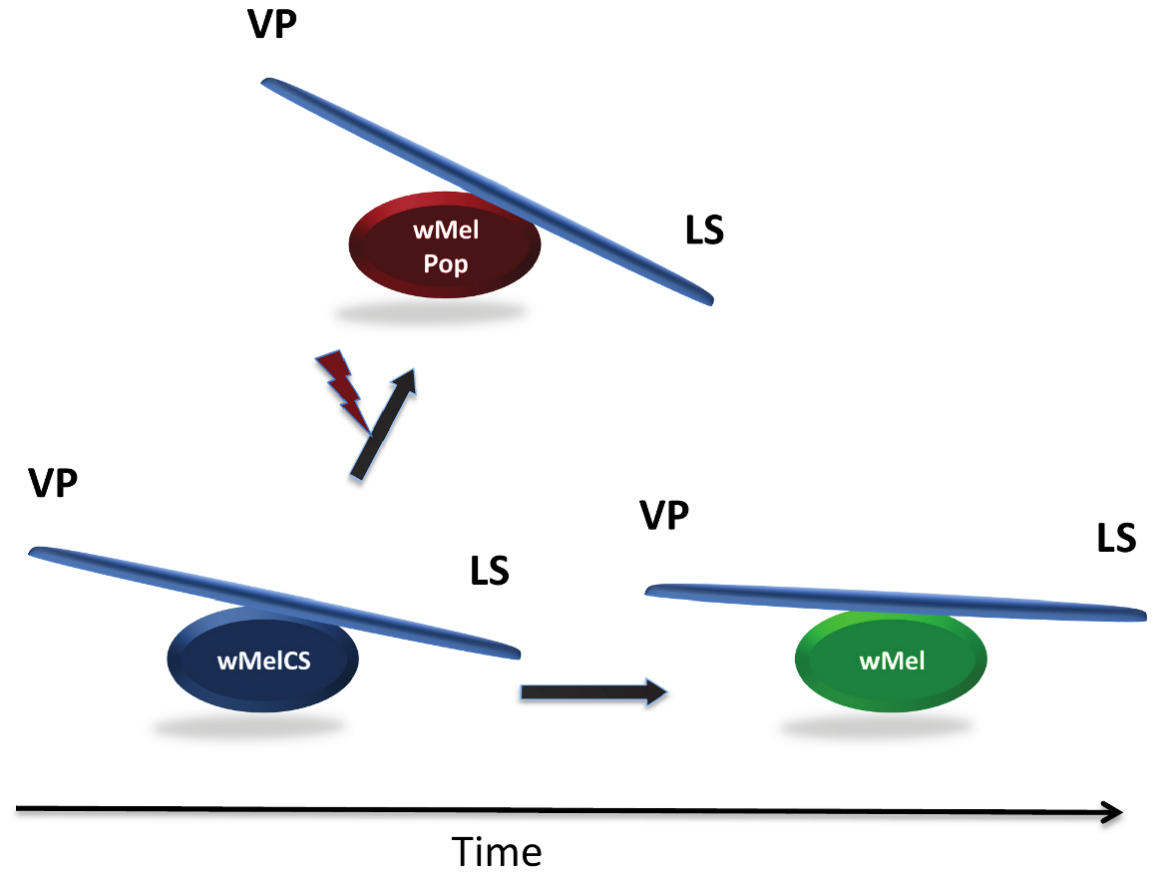
Evolutionary cost–benefit dynamics of *Wolbachia* in natural populations (below) and laboratory lines (above) of *Drosophila melanogaster*. While beneficial *Wolbachia* phenotypes such as virus protection (VP) enhance host fitness, increased *Wolbachia* densities are costly by decreasing lifespan (LS). The ancestral *Wolbachia* variant *w*MelCS is shown in blue, the laboratory-derived pathogen *w*MelPop that has been generated by irradiation (red flash) in red, and the recent worldwide infection variant *w*Mel in green.

To this end, Texeira and colleagues studied the molecular and phenotypic basis of short-term *Wolbachia* dynamics in *D. melanogaster*. First, they crossed the three *Wolbachia* variants wMelCS, wMel, and wMelPop into a common genetic fly background in order to exclude any nuclear background effects. Then they carefully assayed for their capacity to suppress viral infections of the Drosophila C virus and Flock house virus in the presence and absence of *Wolbachia*. They found that the ancestral wMelCS variants proliferate to higher densities and confer greater protection against the two RNA viruses than do flies infected with wMel variants. They also found that flies carrying the ancestral wMelCS infection, although more protected against viral infections, pay a price by living slightly shorter lives than wMel-infected flies (Figure 1). They therefore propose that the ancestral high-cost strain wMelCS (life-shortening due to high titer) has been replaced in nature recently by the less costly *Wolbachia* variant wMel, which still protects the host significantly from viral infections but replicates at lower densities with lower costs on longevity.

Finally, they trace the genetic basis for the transition from symbiosis to virulence by linking phenotype to genotype in the monophyletic wMelCS *Wolbachia* group. Through their thoughtful comparative analysis of mutualistic wMelCS-like versus pathogenic wMelPop genomes, they found that these two phenotypically distinct *Wolbachia* strains form a monophyletic group that mainly differ in only one genomic region: a 7-fold amplification of a 21-kb region in wMelPop encoding eight *Wolbachia* proteins. These data strongly imply that the selective amplification of one or more genes within this so-called “Octomom region” is quite likely responsible for the expression of the virulent Popcorn phenotype.

This impressive study opens numerous additional questions that will be highly relevant for symbiosis research but also for future applied aspects: Which of the eight candidate genes of the Octomom region of wMelPop are sufficient for triggering *Wolbachia* pathogenicity? What are the genetic bases for symbiont-directed viral host protection and its strength? And finally, what kind of phenotypic and genetic *Wolbachia* transitions can be expected to happen in novel host systems such as mosquitos in the near future under natural selection? Stay tuned! It is likely that we are pretty close to monitoring short-term evolutionary dynamics of host–symbiont interactions in real time.
